# Skin necrosis at both COVID-19 vaccine injection sites

**DOI:** 10.1016/j.jdcr.2021.07.022

**Published:** 2021-07-27

**Authors:** Diana Gruenstein, Jacob Levitt

**Affiliations:** Department of Dermatology, Icahn School of Medicine at Mount Sinai, New York, New York

**Keywords:** COVID-19, COVID-19 vaccine, eschar, Pfizer, injection-site reaction, local skin reaction, lymphoma, marginal cell lymphoma, necrosis, skin necrosis

## Introduction

Delayed injection-site reactions (occurring 8 days or more after vaccination) are seen with the COVID-19 vaccine. Typically, the reaction is characterized by pain, erythema, and induration near the injection site that resolves within 5 days.^1^ We report a case of necrotic eschars appearing one week after the second dose of the COVID-19 BNT162b2 (Pfizer) vaccine at the injection sites.

## Case report

**Fig 1:**
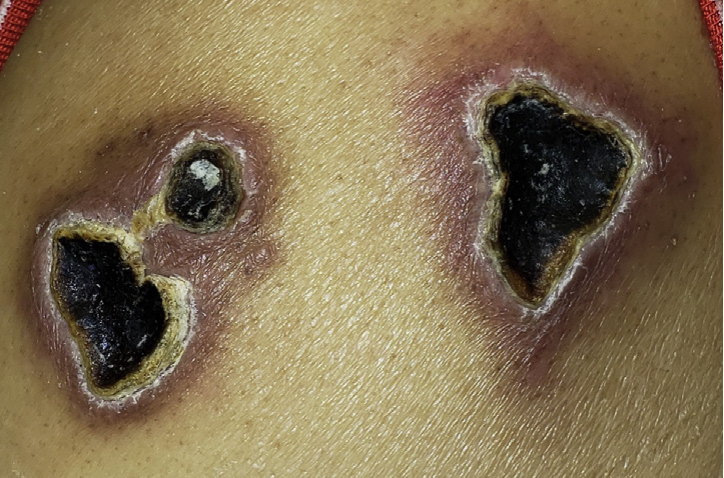
Three angulated eschars of the left shoulder with circumferential indurated erythematous plaques.

A 62-year-old man with hypertension, diabetes mellitus, epilepsy, and stage IV marginal zone B-cell lymphoma with bone marrow involvement on lenalidomide and rituximab presented with 2 necrotic lesions on his left shoulder for 1 month's duration at the 2 injection sites of his 2 BNT162b2 (Pfizer) COVID-19 vaccine. Within 1 week of his second vaccine dose, his left shoulder became red, progressing to bullae and then to necrotic plaques. Complete blood count revealed pancytopenia, with a white blood cell count of 2.1 × 10^3^/uL, hemoglobin of 6.3 G/dL, and platelet count of 9 × 10^3^/uL, all unchanged from baseline. The patient had been receiving platelet transfusions every 1 to 4 weeks. Physical examination revealed 3 angulated eschars of the left deltoid shoulder with surrounding indurated erythematous borders (Fig 1). The clinical picture was consistent with an injection-site reaction. Biopsy was deferred due to the patient's thrombocytopenia. After 2.5 months of no intervention, the lesions were granulating and shrinking but have yet to fully re-epithelialize.

## Discussion

The COVID-19 vaccine is known to cause injection-site reactions characterized by pain, redness, and swelling. The clinical trial by Baden et al^1^ for the mRNA-1273 (Moderna) vaccine found immediate injection-site reactions in 84.2% of participants and delayed injection-site reactions in 0.8% and 0.2% of patients after their first and second dose, respectively. The delayed injection-site reactions typically resolved within 4 to 5 days after first appearing. The pathophysiology of the delayed injection-site reactions involves delayed-type T-cell mediated hypersensitivity reactions, supported by superficial perivascular and perifollicular lymphocytic infiltrate with rare eosinophils and scattered mast cells on biopsy.^2^

Weinstein-Guttman et al reported 1 case of delayed injection-site reaction (7 days) after both doses of BNT162b2 in a patient with hematological and neuroinflammatory comorbidities who was on rituximab, similar to our patient who had hematological comorbidities and was on rituximab. The patient developed diffuse urticarial papules and plaques predominately on the left side of her body, the same side in which she was vaccinated. The lesions resolved with a 3-week course of corticosteroids.^3^

The case reported here is unique in that the reaction appeared to be thrombotic in nature, with a morphology similar to the retiform purpura of calciphylaxis. COVID-19 disease itself is characterized by thrombosis, thought to be due to increased angiotensin II and tissue factor.^4^ The Ad26.COV2.S (Johnson & Johnson) 1-dose vaccine was rarely associated with thrombosis with thrombocytopenia syndrome. The pathomechanism could be similar to heparin-induced thrombocytopenia given that the heparin platelet-4 heparin-induced thrombocytopenia antibody enzyme-linked immunosorbent assay was positive in a majority of the affected patients.^5^ Thrombotic complications occur in only a minuscule subset of vaccine recipients and perhaps in only a minority of COVID-19 cases, typically the more severe ones. This may perhaps predict a genetic susceptibility to this phenomenon. Alternatively, our patient's underlying malignancy and baseline thrombocytopenia may account for this atypical presentation, which is unlikely to occur in the general population.

Given the mass scale of COVID-19 vaccinations occurring globally, clinicians will likely see many patients who complain of both immediate and delayed injection-site reactions. Most reactions will be mild and be characterized by pain, edema, and redness; however, some reactions, as seen in our patient, can be severe. Nevertheless, the average person will not experience such reactions, and the risk-benefit ratio overwhelmingly favors vaccination in spite of these rare, often inconsequential (with rare exception) vaccine reactions.

## Conflicts of interest

Dr Levitt has served on Advisory Boards for UCB, Leo Pharma, Novartis, AbbVie, and Arcutis Biotherapeutics. Dr Levitt has been a Consultant for Novartis and AbbVie. Dr Gruenstein has no conflict of interest to declare.
